# NLRP3 Gene Silencing Ameliorates Diabetic Cardiomyopathy in a Type 2 Diabetes Rat Model

**DOI:** 10.1371/journal.pone.0104771

**Published:** 2014-08-19

**Authors:** Beibei Luo, Bo Li, Wenke Wang, Xiangjuan Liu, Yanfei Xia, Cheng Zhang, Mingxiang Zhang, Yun Zhang, Fengshuang An

**Affiliations:** The Key Laboratory of Cardiovascular Remodeling and Function Research, Chinese Ministry of Education and Chinese Ministry of Health, Shandong University Qilu Hospital, Jinan, Shandong, China; University of Pittsburgh School of Medicine, United States of America

## Abstract

**Background:**

Nucleotide-binding oligomerization domain-like receptor protein 3 (NLRP3) inflammasome is associated with metabolic disorder and cell death, which are important triggers in diabetic cardiomyopathy (DCM). We aimed to explore whether NLRP3 inflammasome activation contributes to DCM and the mechanism involved.

**Methods:**

Type 2 diabetic rat model was induced by high fat diet and low dose streptozotocin. The characteristics of type 2 DCM were evaluated by metabolic tests, echocardiography and histopathology. Gene silencing therapy was used to investigate the role of NLRP3 in the pathogenesis of DCM. High glucose treated H9c2 cardiomyocytes were used to determine the mechanism by which NLRP3 modulated the DCM. The cell death in vitro was detected by TUNEL and EthD-III staining. TXNIP-siRNA and pharmacological inhibitors of ROS and NF-kB were used to explore the mechanism of NLRP3 inflammasome activation.

**Results:**

Diabetic rats showed severe metabolic disorder, cardiac inflammation, cell death, disorganized ultrastructure, fibrosis and excessive activation of NLRP3, apoptosis-associated speck-like protein containing a caspase recruitment domain (ASC), pro-caspase-1, activated caspase-1 and mature interleukin-1β (IL-1β). Evidence for pyroptosis was found *in vivo*, and the caspase-1 dependent pyroptosis was found *in vitro*. Silencing of NLRP3 *in vivo* did not attenuate systemic metabolic disturbances. However, NLRP3 gene silencing therapy ameliorated cardiac inflammation, pyroptosis, fibrosis and cardiac function. Silencing of NLRP3 in H9c2 cardiomyocytes suppressed pyroptosis under high glucose. ROS inhibition markedly decreased nuclear factor-kB (NF-kB) phosphorylation, thioredoxin interacting/inhibiting protein (TXNIP), NLRP3 inflammasome, and mature IL-1β in high glucose treated H9c2 cells. Inhibition of NF-kB reduced the activation of NLRP3 inflammasome. TXNIP-siRNA decreased the activation of caspase-1 and IL-1β.

**Conclusion:**

NLRP3 inflammasome contributed to the development of DCM. NF-κB and TXNIP mediated the ROS-induced caspase-1 and IL-1β activation, which are the effectors of NLRP3 inflammasome. NLRP3 gene silencing may exert a protective effect on DCM.

## Introduction

Diabetic cardiomyopathy (DCM), characterized by consistent diastolic dysfunction and increased ventricular mass, is the leading cause of mortality among patients with diabetes [1], [2]. Hyperglycemia-induced reactive oxygen species (ROS) generation is considered to be responsible for progression and development of DCM [3], [4]. The increased ROS could induce a number of cytokine and inflammatory factors, such as nuclear factor-kB (NF-kB), thioredoxin interacting/inhibiting protein (TXNIP), and inflammasome [5], [6], [7]. Although inflammasome was shown to be involved in the pathogenic mechanisms of type 2 diabetes and its complications [8], [9], the potential role and regulatory mechanism of inflammasome in DCM has remained largely unexplored.

Inflammasomes are multi-protein platforms that interact with various immune and cell death pathways [10], [11]. Different inflammasomes have been identified, including nucleotide-binding oligomerization domain-like receptors (NLRs) and absent in melanoma 2 (AIM2) [12]. NLRP3, the most extensively studied NLRs, forms a complexes comprised of the apoptosis associated speck like protein (ASC), and the serine protease caspase-1 [13]. On activation, NLRP3 forms a complex with its adaptor ASC, which facilitates the autocatalytic activation of pro-caspase-1 and the formation of an active caspase-1 p10/20 tetramer [11]. The activated caspase-1 can process pro-IL-1β into its mature form, which is important in cardiomyocyte apoptosis [11], [14].

In addition to resulting in the maturation of IL-1β, activated caspase-1 can induce a distinct form of programmed cell death called “pyroptosis” [15]. Pyroptosis, a highly inflammatory form of cell death, is dependent on caspase-1 activity [16]. The morphology of pyroptosis shares the unique characteristics with both apoptosis and necrosis [15]. As in apoptotic cell, pyroptotic cells incur DNA damage and become positive in the terminal deoxynucleotidyl transferase-mediated dUTP nick end-labeling (TUNEL) staining. Similar to necrosis, pyroptosis results in pore formation in the cell membrane, release of pro-inflammatory cytosolic content, and cell lysis. Therefore, membrane impermeant dyes such as EthD-III stain pyroptotic cells by entering through the pores, but do not stain apoptotic cells [17], [18]. Pyroptosis is initially described in macrophages and dendritic cells infected with different pathogens [19], [20]. Recent studies showed that pyroptosis could also occur in non-myeloid cells induced by non-infectious stimuli [21], [22], [23]. Electron microscopy studies of myocardium in diabetic mice and rats showed that the majority of dying cells had swollen fibril and mitochondria, which are the characteristics of cell swelling and lysis in pyroptosis [24], [25], [26]. Activated caspase-1, the executor caspase of pyroptosis, is found to be elevated in DCM in a rat model. However, it is not clear whether pyroptosis participates in hyperglycemia-induced cardiomyocyte death.

Recent studies indicate that NF-kB mediated the ROS-induced NLRP3 inflammasome by promoting the transcription of NLRP3 inflammasome [27], [28]. Thioredoxin interacting/inhibiting protein (TXNIP) can bind NLRP3 directly and lead to NLRP3 inflammasome assembly under oxidative stress [6], [29]. However, little is known about whether NF-κB and TXNIP participate in the regulation of NLRP3 in hyperglycemia-treated cardiomyocyte.

In this study, we hypothesized that pyroptosis, regulated by NLRP3 inflammsome, might participate in the pathogenesis of DCM. We also hypothesized that NF-κB and TXNIP might be links between ROS and NLRP3 inflammasome activation.

## Materials and Methods

### Animal study

**Figure 1:**
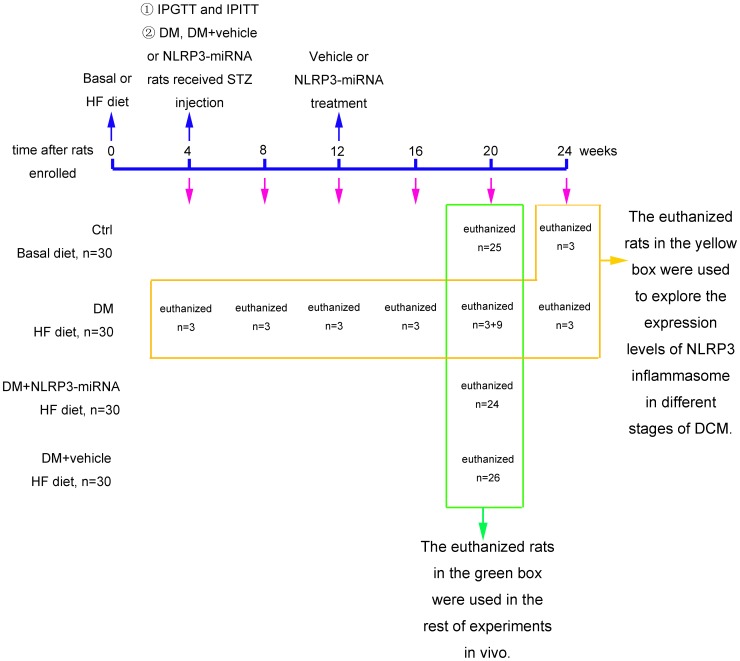
The information of experimental design. The euthanized rats in the yellow box were used to explore the expression levels of NLRP3 inflammasome in different stages of DCM. The sacrificed rats in the green box were used in the rest of experiments in vivo. IPGTT: intraperitoneal glucose tolerance test; IPITT: intraperitoneal insulin tolerance test; HF: high fat diet; Ctrl: control rats; DM: diabetes rats; DM+NLRP3-miRNA: diabetes rats received lentiviral NLRP3-miRNA; DM+vehicle: diabetes rats received empty lentiviral vector.

We randomized 120 Sprague-Dawley rats (100–120 g) into 4 groups (n = 30 per group): control, diabetes mellitus (DM), DM + vehicle, DM + NLRP3-miRNA. All rats were housed at 22°C with 12 h light-dark cycles. The control group was fed the basal diet, and the other groups a high fat diet (HF diet, 16% fat and 0.25% cholesterol). 4 weeks later, the intraperitoneal glucose tolerance test (IPGTT) and intraperitoneal insulin tolerance test (IPITT) were performed to determine insulin resistance. Diabetes was induced by a single intraperitoneal injection of streptozotocin (STZ; 35 mg/kg, Solarbio, China) to rats in the groups of DM, DM + vehicle and DM + NLRP3-miRNA. After one week of STZ injection, blood glucose levels were measured (Roche, Germany) after rats fasted overnight. Only rats with blood glucose level ≥11.1 mmol/L were used in the study. According to previous studies, diabetic rats showed onset of cardiac dysfunction after 8 weeks of STZ injection [30], [31]. Our gene silencing treatment occurred after 8 weeks of STZ injection (Fig. 1). The groups of DM + vehicle and DM + NLRP3-miRNA rats in our study received a jugular-vein injection of a total lentivector dose of 1×10^8^ TU/rat vehicle (empty virus) or NLRP3-miRNA (Invitrogen, Shanghai, China). Sequences for rat NLRP3-miRNA oligos were: forward, 5′- TGCTGATAAGAAGTTCTCTCCTGGTTGTTTTGGCCACTGACTGACAACCAGGAGAACTTCTTAT-3′; and reverse, 5′- CCTGATAAGAAGTTCTCCTGGTTGTCAGTCAGTGGCCAAAACAACCAGGAGAGAACTTCTTATC -3′. After 8 weeks of lentivector injection, treated rats and age-matched control and DM rats were killed. The heart was excised and immediately frozen to determine the transfection efficiency under fluorescence microscope.

For the analysis of NLRP3 inflammasome and IL-1β expression, cardiac samples of DM rats were collected at 0, 4, 8, 12, 16 and 20 weeks (n = 3) after STZ injection, and control rats (n = 3) were collected at 20 weeks after diabetic rats received STZ (Fig. 1). The experiments complied with the Animal Management Rule of Chinese Ministry of Health (documentation no. 55, 2001), and experimental protocols were approved by the Shandong University Animal Care Committee.

### Serum measurements

After rats fasted overnight, serum triglycerides (TG) and total cholesterol (TC) were determined by use of an automatic analyzer (Roche, Basel, Switzerland). Serum level of insulin was measured by enzyme-linked immunosorbent assay (Jiancheng, Nanjing, China).

### Echocardiography

Transthoracic echocardiography involved the Vevo 770 imaging system with RMB710 transducer (VisualSonics, Toronto, Canada). Images were obtained from rats anesthetized with 10% chloral hydrate. The derived echocardiography parameters included left ventricular end-diastolic dimension (LVEDd), left ventricular ejection fraction (LVEF), fractional shortening (FS), peak E to peak A ratio (E/A), and early (E′) to late (A′) diastolic velocity ratio (E′/A′).

### Histological examination

The hearts were arrested with 10% KCl, and heart weight was measured immediately. The images of whole heart and cross sections from the midpoint of heart were obtained by camera (Canon, Tokyo, Japan).

### Measurement of myocardial fibrosis

Masson's trichrome and Sirius red staining were used for detecting collagen. The fibrotic area fraction was analyzed by automated image analysis (Image-Pro Plus, Media Cybemetics, US).

### Transmission electron microscopy (TEM)

About 1 mm^3^ tissue was obtained from the left ventricle of rats and fixed in 2.5% glutaraldehyde in 0.1 mol/L sodium cacodylate buffer (pH 7.4) for 2 h. The following steps were as described previously [32]. Finally, the specimens were studied by transmission electron microscopy (TEM, JEM-1200EX, Japan).

### Immunohistochemistry

Immunohistochemistry was as described previously [33]. We used antibodies against caspase-1 (1∶50, Abcam, UK) and IL-1β (1∶200). Data were analyzed by Image-Pro Plus 6.0 (Media Cybemetics, US).

### Real-time RT-PCR

Real-time RT-PCR was as described previously [34]. The primer sequences are shown in Table 1. The relative expression of genes was calculated by the 2^−ΔΔCT^ method.

**Table 1:** Primer sequences for real-time RT-PCR.

Symbol	Forward primer	Reverse primer
NF-kB	GAGATTGTGCCAAGAGTGAC	CTTGTCTTCCATGGTGGATG
TXNIP	GCTCAATCATGGTGATGTTCAAG	CTTCACACACTTCCACTGTCAC
NLRP3	GTGGAGATCCTAGGTTTCTCTG	CAGGATCTCATTCTCTTGGATC
ASC	CTCTGTATGGCAATGTGCTGAC	GAACAAGTTCTTGCAGGTCAG
Caspase-1	GAGCTGATGTTGACCTCAGAG	CTGTCAGAAGTCTTGTGCTCTG
IL-1β	TGCTGTCTGACCCATGTGAG	GTCGTTGCTTGTCTCTCCTTG
β-actin	AGACCTTCAACACCCCAG	CACGATTTCCCTCTCAGC

TXNIP, thioredoxin interacting/inhibiting protein; NLRP3, Nucleotide-binding oligomerization domain-like receptor protein 3; ASC, apoptosis-associated speck-like protein containing a caspase recruitment domain; IL-1β, interleukin-1β.

### Western blot

Western blot was as described previously [34]. We used antibodies against NF-κB (1∶700, t-NF-KB p65 ab131485, p-NF-kB p65, ab28856, Abcam, UK), TXNIP (1∶700, ab86983), NLRP3 (1∶500, ab109314), ASC (1∶700, ab64808), caspase-1 (1∶500, ab1872), IL-1β (1∶1000, ab9722), collagen I (1∶1000, ab34710), collagen III (1∶500, ab7778), and β-actin (1∶1000, sc8432, Santa Cruz, USA). The protein bands were developed by the use of chemiluminescence (Millipore, USA), and quantified by densitometric analysis (Quantity One, Bio-Rad, USA).

### TUNEL assay

Detection of DNA cleavage was performed using an ApopTag in situ apoptosis detection kit (Millipore, USA). Deparaffinized sections were treated with 3% hydrogen peroxide in methanol for 10 min. After adding the equilibration buffer, sections were treated with TdT-enzyme at 37°C for 1 h. The sections were then incubated with digoxigenin-conjugated antibodies at 37°C for 30 min. Then sections were colorized with DAB (ZSGB-BIO, China). Finally, the stained sections were examined under microscope (Olympus, Japan).

### Cell culture and treatment

H9c2 cardiomyocytes culture was as described previously [35]. During the treatment period, H9c2 cells were cultured in normal glucose medium with minimal essential medium for 12 h, followed by the exposure of control glucose (Ctrl, 5.5 mM), medium glucose (MG, 25 mM), high glucose (HG, 33.3 mM), and osomotic control (OC, 27.5 mM mannose) for 24, 36, or 48 h. In ROS inhibition experiment, cells were treated with HG for 36 h in the presence of 10 mmol/L N-acetylcysteine (NAC, Beyotime, China) or PBS. For NF-kB inhibition, cells were pretreated with 10 µmmol/L BAY 11-7082 or DMSO before HG stimulation.

### Lentivirus transfection

H9c2 cardiomyocytes plated at a density of 1×10^5^/cm^2^ were infected with vehicle or lentivrius-NLRP3-miRNA at 10 multiplicity of infection (MOI) in serum-free media for 2 h, followed by incubation with DMEM containing 10% FBS for an additional 48 h before processing. The transfection efficiency was observed by fluorescence microscope. For each experiment, NLRP3 knockdown was assessed in transfected cells, and cells were used only if NLRP3 mRNA was decreased by 70% compared with vehicle.

### siRNA transfection

H9c2 cardiomyocytes were transfected with vehicle (empty plasmid) or TXNIP-siRNA plasmid (JiMa, Shanghai, China) by using Lipofectamin 2000 (Invitrogen, Shanghai, China). Sequences for rat TXNIP-siRNA were: sense, 5′- UGGUCACGUCGAAAUGAAUTT-3′; antisense, 5′-TTACCAGUGCAGCUUUACUUA-3′.

### Immunofluorescence

Immunofluorescence was as described previously [36]. We used antibodies against caspase-1 (1∶50). The fluorescence was visualized by confocal microscopy (LSM710, Carl Zeiss, Germany) and analyzed by Image-Pro Plus 6.0 (Media Cybemetics, US).

### Caspase-1 activity assay

Caspase-1 activity was measured by using colorimetric assay (Beyotime, China). This assay was based on the ability of caspase-1 to change acetyl-Tyr-Val-Ala-Asp p-nitroaniline (Ac-YVAD-pNA) into the yellow formazan product p-nitroaniline (pNA). 50 ug of total cytosolic protein was incubated in a 96-well microtiter plate with 20 nmol Ac-YVAD-pNA overnight at 37°C. The absorbance values of pNA at 405 nm, OD_405_, were tested using spectrophotometer Varioscan Flash and SkanIt Software Version 2.4.3. RE (Thermo Fisher Scientific Inc, USA). A standard curve of pNA was performed. The production of pNA in tested samples indicated the level of caspase-1 activation.

### Enzyme-linked immunosorbent assay (ELISA)

IL-1β, IL-18, TNF-α and IL-6 in cultured cardiomyocyte supernatants were measured using commercial ELISA kits (Jiancheng, Nanjing, China).

### ROS levels

Intracellular generation of ROS was tested by peroxide-sensitive fluorescent probe 2′,7′-dichlorofluorescein diacetate (DCFH-DA, Sigma, Shanghai, China). The stimulated cells were washed twice with cold PBS and incubated with DCFH-DA at 37°C for 30 min in dark. Fluorescent signal of 6 fields per groups was recorded by using a fluorescence microscopy (488 nm filter, Olympus, Japan). Fluorescence intensity was analyzed with HMIAS-2000 software.

### Cell death assay

Cell death was assessed by TUNEL assay and EthD-III/calcein AM staining. For TUNEL assay, cells cultured in chamber slide were fixed with 4% paraformaldehyde for 30 min. Cells were permeabilized with immunol staining wash buffer (Beyotime, China) for 2 min on ice. The following steps were as introduced above. EthD-III/calcein AM staining (Viability/Cytotoxicity Assay Kit, Biotium, USA) was also used for cell death assay. The AM produces a bright green fluorescence in live cells. EthD-III enters dead cells, thereby producing red fluorescence in dead cells. Cells were simultaneously stained with 2 µM calcein AM and 4 µM EthD-III at 37°C for 45 min. Then cells were incubated with Hoechst 33342 (Beyotime, China) at 37°C for 30 min. Coverslips were finally sealed by a drop of antifade mounting medium (Beyotime, China). The fluorescence was visualized by confocal microscopy (LSM710, Carl Zeiss, Germany). The percentage of dead cell was achieved by counting the ratio of red positive cells to of blue positive cells.

### Statistical analysis

Data are expressed as mean±SEM. Differences among experimental groups were analyzed by ANOVA, followed by the Tukey-Kramer post hoc test and independent samples t test. Analysis involved use of SPSS v18.0 (SPSS, Chicago, IL, USA). Significance was defined as p<0.05.

## Results

### NLRP3 inflammasome and IL-1β were activated in DCM

**Figure 2:**
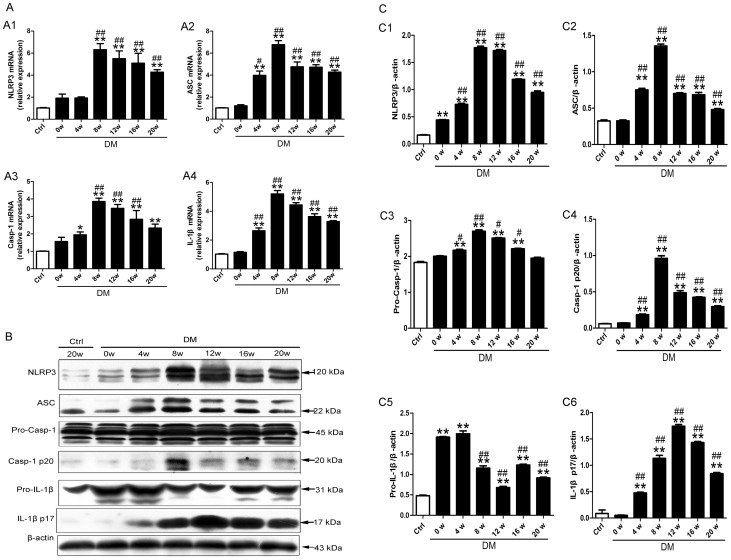
NLRP3 inflammasome was activated in myocardium after 4 weeks of diabetes. (A) The relative mRNA expression of NLRP3 (A1), ASC (A2), caspase-1(A3) and IL-1β (A4). (B and C) The protein levels of NLRP3 (C1), ASC (C2), pro-caspase-1 (C3), activated caspase-1(C4), pro-IL-1β (C5) and mature IL-1β (C6). Data were presented as means±SEM, n = 3. *p<0.05, **p<0.01 vs. Ctrl at 20 w; ^#^p<0.05, ^##^p<0.01 vs. DM at 0 w. Ctrl: control rats sacrificed at 20 weeks after diabetes rats received STZ injection; DM: diabetes rats; w: weeks; Pro-Casp-1: pro-caspase-1; Casp-1 p20: activated caspase-1; IL-1β p17: mature IL-1β.

When compared with the control rats, diabetic rats showed elevated mRNA and protein levels of NLRP3, ASC, caspase-1 and IL-1β beginning at 4 or 8 weeks of diabetes (Fig. 2A–C, all p<0.01). Moreover, the mRNA and protein levels of NLRP3 inflamamsome components and IL-1β achieved the highest levels at 8 or 12 weeks of diabetes except pro-IL-1β (Fig. 2A–C, all p<0.01).

### NLRP3 gene silencing improved metabolism abnormalities

In the DM group, blood glucose, TC, TG and INS were higher than the control (Table 2, all p<0.01). ISI in DM group was lower than control group (Table 2, p<0.01). NLRP3 gene silencing was insufficient to improve the systemic metabolism disorder.

**Table 2:** NLRP3 gene silencing did not attenuate metabolic abnormalities.

	Ctrl	DM	DM + Vehicle	DM + NLRP3-miRNA
Blood glucose (mmol/L)	5.08±0.38	18.80±0.39**	18.55±0.65**	18.51±0.82**
TC (mmol/L)	1.63±0.05	2.70±0. 08**	2.73±0.09**	2.71±0.04**
TG (mmol/L)	0.65±0.06	2.72±0.08**	2.71±0.15**	2.67±0.05**
INS (mmol/L)	14.10±0.34	16.19±0.12**	16.09±0.12**	16.13±0.34**
ISI	−4.26±0.09	−5.71±0.02**	−5.69±0. 04**	−5.68±0. 05**

The blood samples were collected at the end of the experiment. Ctrl, control; DM, diabetes mellitus; Blood glucose, blood glucose tested at the end of the experiment; TG, triglyceride; TC, total cholesterol; INS, insulin tested at the end of the experiment; ISI, insulin sensitivity index. Data are mean±SEM, n = 6–8 per group.

**p<0.01 vs. control.

### Cardiac NLRP3 expression was suppressed by gene silencing

**Figure 3:**
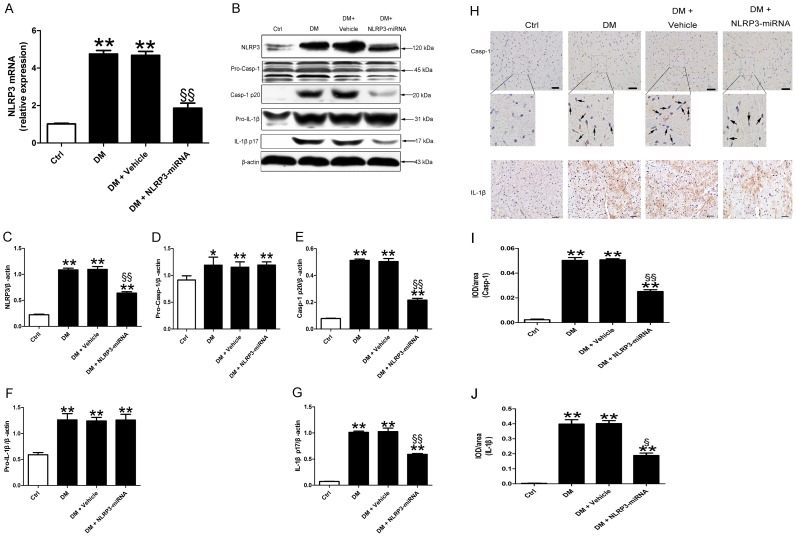
The inhibition of NLRP3 was effective in vivo. Relative mRNA expression of NLRP3 (A) and protein levels of NLRP3, pro-caspase-1, activated caspase-1, pro-IL-1β and mature IL-1β (B–G) in cardiac tissue with NLRP3-miRNA treatment. Immunohistochemical staining (H) and a semi-quantitative analysis of positive staining (I–J) of caspase-1 and IL-1β; scale bar: 20 µm. Data were presented as means±SEM, n = 6. **p<0.01 vs. Ctrl; ^§^p<0.05, ^§§^p<0.01 vs. DM+vehicle. Ctrl: control rats sacrificed at 16 weeks after diabetes rats received STZ injection; DM : diabetes rats sacrificed at 16 weeks after STZ injection; Casp-1 p20: activated caspase-1; IL-1β p17: mature IL-1β.

After 8 weeks of NLRP3 silencing treatment, transfection efficiency was checked in all groups (Fig. S1A). As compared with vehicle treatment, NLRP3-miRNA treatment decreased the mRNA and protein levels of cardiac NLRP3 (Fig. 3A–C, both p<0.01). In addition, the protein levels of activated caspaspe-1 and mature IL-1β were lower in NLRP3-miRNA treated diabetic rats than the vehicle treated rats (Fig. 3B, E and G, both p<0.01). NLRP3-miRNA did not have effect on the protein levels of pro-caspase-1 and pro-IL-1β (Fig. 3B, D and F). Immunohistochemistry revealed that increased caspase-1 predominantly localized in perinuclear area, while elevated IL-1β showed diffused distribution pattern in DCM (Fig. 3H–J, p<0.05∼p<0.01).

### NLRP3 gene silencing alleviated left ventricular dysfunction in DCM

**Figure 4:**
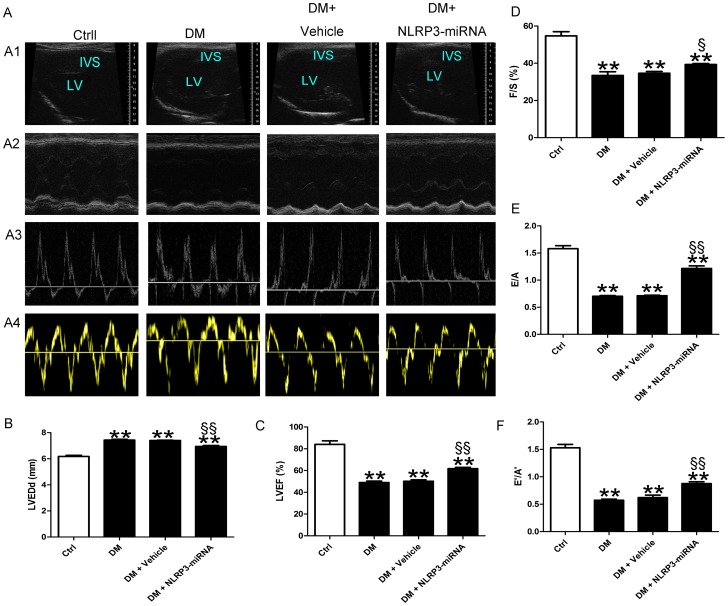
NLRP3 gene silencing improved cardiac dysfunction in diabetes rats. (A) The cardiac function of rats in different groups shown by echocardiography. Representative images of 2D echocardiograms (A1), M-mode echocardiograms (A2), pulse-wave Doppler echocardiograms of mitral inflow (A3), tissue Doppler echocardiograms (A4). (B–F) Evaluation of LVEDd, LVEF, FS, E/A and E′/A′. Data were presented as means±SEM, n = 6. **p<0.01 vs. Ctrl; ^§^p<0.05, ^§§^p<0.01 vs. DM+vehicle. LV: left ventricle; IVS: interventricular septum; LVEDd: left ventricular end-diastolic dimension; LVEF: left ventricular ejection fraction; FS: fractional shortening; E/A: peak E to peak A ratio; E′/A′: early (E′) to late (A′) diastolic velocity ratio.

The results of echocardiography showed that LVEDd of DM rat was larger than control (Fig. 4A and B, p<0.01). LVEF, FS, E/A and E′/A′ were lower in DM than control (Fig. 4A, C–F, both p<0.01). When compared with vehicle, increased LVEF, FS, E/A, E′/A′ and decreased LVEDd were observed in NLRP3-miRNA group (Fig. 4A–F, p<0.05∼p<0.01).

### NLRP3 gene silencing reversed myocardial remodeling

**Figure 5:**
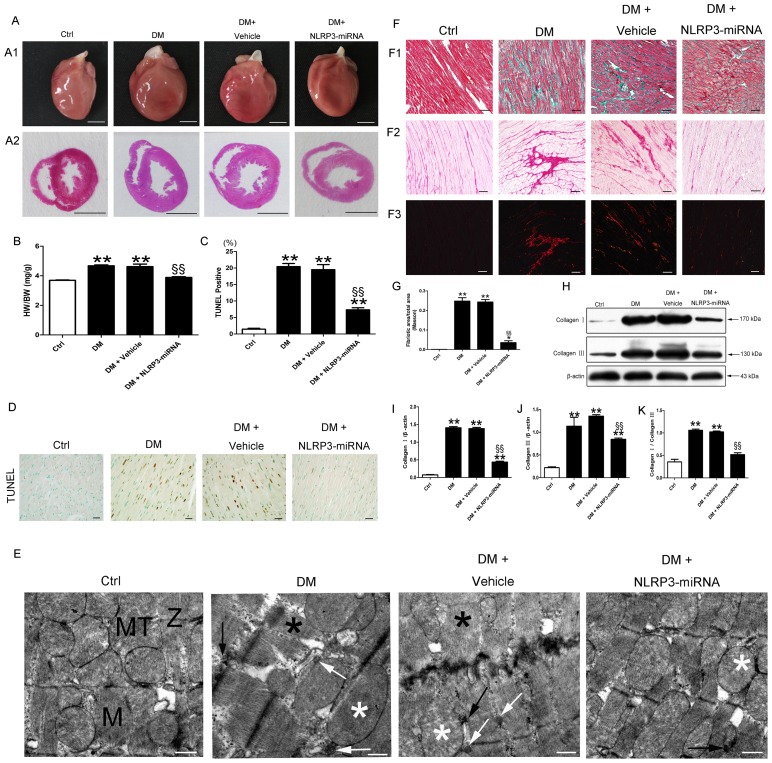
NLRP3 gene silencing alleviated myocardial cell death, ultrastructral disorder and fibrosis in diabetic group. Heart size (A1) and cross sections of hearts at the papillary muscle level (A2); scale bar: 5 mm. (B) Quantitative data for heart weight to body weight ratio. TUNEL staining of myocardium (D, scale bar: 20 µm) and quantitative analysis of TUNEL positive cells (C) in each group. (E) Transmission electron microscopy of rat hearts; normal myofibrils (M); normal mitochondria (MT); normal Z-lines (Z); disorganized myofibrils (black asterisk); swollen mitochondria (white asterisk); accumulated lipid (white arrow); excessive glycogen lysis (black arrow); scale bar: 1 µm. (F) Cardiac fibrosis in rats; (F1) Masson staining; fibrotic areas stained green and normal cardiac myocytes stained red; bright field (F2) and dark field (F3) with Sirius red staining; collagen was stained dark red in bright field; collagen I was stained red and collagen III green in dark field; scale bar: 50 µm. (G) Quantitative analysis of the fibrotic area to total area ratio. (H–K) Western blot analysis of collagen I and III. Data were presented as means±SEM, n = 4–6. **p<0.01 vs. Ctrl; ^§§^p<0.01 vs. DM+vehicle.

The DM group showed the phenotype of eccentric ventricular hypertrophy (Fig. 5A). The ratio of heart weight to body weight was larger for DM than the control group (Fig. 5B, p<0.01). TUNEL result showed that the percentage of dead cell was higher in DM group than control (Fig. 5C and D, p<0.01). The ultrastructure of cardiomyocyte in control rats showed typical symmetric myofibrils, well-organized Z lines with sarcomeres, and packed mictochontria beside the fibers (Fig. 5E). In contrast, DM rats showed severe damage of the left ventricular ultrastructure, including destruction of myofibrils, swollen mitochondria with disorganized cristae, excess glycogen lysis, and accumulated lipids (Fig. 5E). Moreover, the diabetic group showed increased interstitial cardiac fibrosis as compared with the control (Fig. 5F and G, p<0.01). Coincident with cardiac fibrosis, the protein levels of collagen I and collagen III, and the collagen I-to-III ratio were higher in DM group than the control (Fig. 5H–K, all p<0.01).

With NLRP3 gene silencing, heart weight to body weight ratio and cell death were decreased in the NLRP3-miRNA group versus the vehicle group (Fig. 5B, C and D, both p<0.01). NLRP3-miRNA treatment normalized alterations in myofilaments and mitochondria, along with reduced glycogen lysis and lipid accumulation in diabetic rats (Fig. 5E). Moreover, cardiac fibrosis area, collagen I, collagen III, and the collagen I to III ratio were lower in the DM + NLRP3-miRNA group than vehicle group (Fig. 5F–K, all p<0.01).

### The increased expression of NLRP3 inflammasome and IL-1β were induced by high glucose

**Figure 6:**
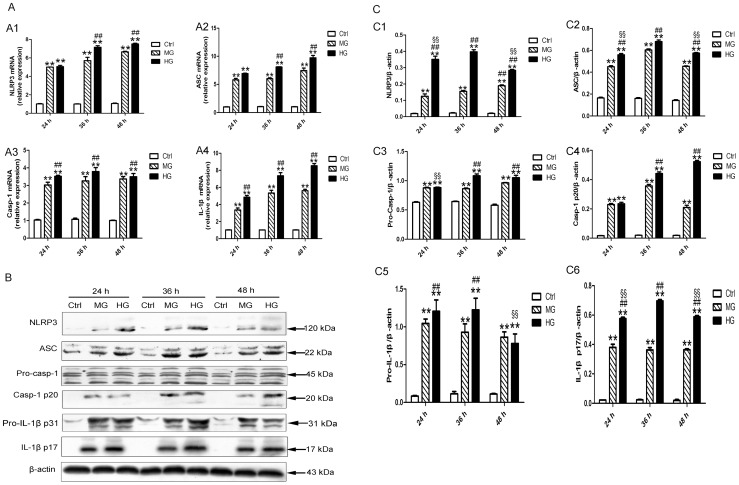
High glucose induced NLRP3 inflammasome and IL-1β expression in H9c2 cardiomyocytes. (A) The relative mRNA expression of NLRP3 (A1), ASC (A2), caspase-1(A3) and IL-1β (A4). (B–C) Western blot analysis of NLRP3 (C1), ASC (C2), pro-caspase-1(C3), activated caspase-1(C4), pro-IL-1β (C5) and mature IL-1β (C6). Data were presented as means±SEM, from 3 independent experiments. The significance among Ctrl, MG and HG within the same stimulated time was analyzed by one way ANOVA. The significance among HG groups within different stimulated time was analyzed by one way ANOVA. **p<0.01 vs. Ctrl in 24 h, 36 h or 48 h; ^##^p<0.01 vs. MG in 24 h, 36 h or 48 h; ^§^p<0.05, ^§§^p<0.01 vs. HG for 36 h. Ctrl: control glucose, 5.6 mM glucose; MG: medium glucose, 25 mM glucose; HG: high glucose, 33.3 mM glucose; Pro-Casp-1: pro-caspase-1; Casp-1 p20: activated caspase-1; IL-1β p17: mature IL-1β.

Different levels of glucose caused a concentration-dependent increase of NLRP3, ASC, caspase-1 and IL-1β mRNA in H9c2 cells in 36 to 48 h (Fig. 6A, all p<0.01). Likewise, the protein levels of all NLRP3 inflammasome components, pro- IL-1β and mature IL-1β were increased at high glucose as compared with control and medium glucose in 36 and 48 h (Fig. 6B and C, all p<0.01). Moreover, the increase of NLRP3, ASC and mature IL-1β protein showed the highest level at 36 h (Fig. 6B, C1, C2 and C6, all p<0.01). Thus, we chose high glucose as the stimulation, and chose 36 h as the stimulated time in subsequent experiments. The expression of NLRP3 inflammasome and mature IL-1β in H9c2 cells with control glucose or isotonic mannose had no significant change at the same time point tested (Fig.S1 B and C).

### High glucose induced caspase-1 activation and cell death in H9c2 cells

**Figure 7:**
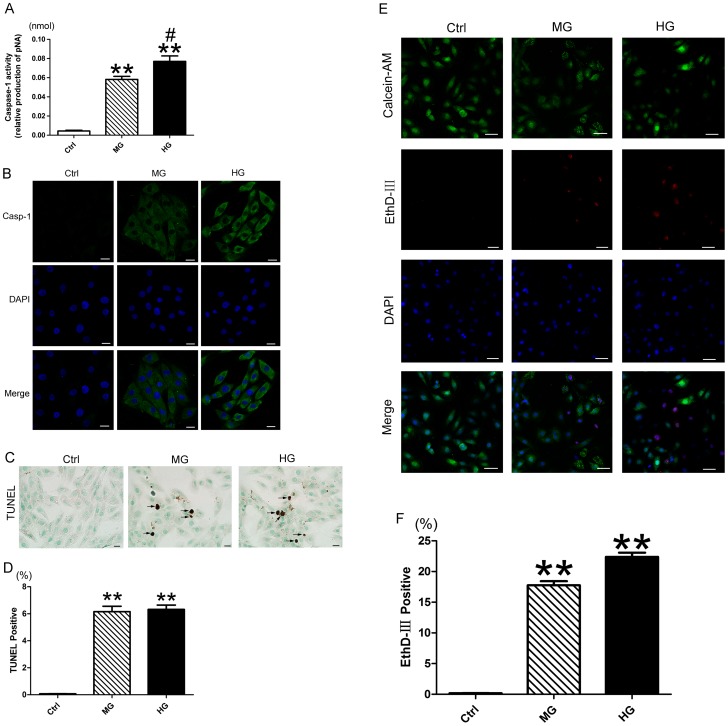
Pyroptosis is implicated in the high glucose-induced cell death in H9c2 cells. (A) Analysis of caspase-1 activity using Ac-YVAD-pNA, the activity of caspase-1 was reflected by the production of pNA. (B) Immunofluorescence analysis of caspase-1in H9c2 cardiomyocytes; scale bar: 20 µm. (C) TUNEL staining of medium and high glucose-treated H9c2 cells, positive cell indicated by black arrow; scale bar: 20 µm. (D) Quantitative analysis of TUNEL positive cell in each group. (E) EthD-III (red) and calcein AM (green) staining; scale bar: 50 µm. (F) Quantitative analysis of EthD-III positive cell. Data were presented as means±SEM, from 3 independent experiments. **p<0.01 vs. Ctrl; ^#^p<0.05 vs. MG. Ctrl: control glucose, 5.6 mM glucose for 36 h; MG: medium glucose, 25 mM glucose for 36 h; HG: high glucose, 33.3 mM glucose for 36 h; Casp-1: activated caspase-1.

High glucose increased the level of activated caspase-1 as compared with control and medium glucose (Fig. 7A, p<0.05∼p<0.01). Immunofluorescence result showed the accumulation of caspase-1 in the cytoplasm of H9c2 cells incubated with medium and high glucose (Fig. 7B). Coincident with caspase-1 activation in high glucose, TUNEL result revealed increased cell death with nucleus DNA damage (Fig. 7C and D, p<0.01), and EthD-III/calceim AM staining showed elevated cell death with damaged cell membrane as compared with control and medium glucose (Fig. 7E and F, p<0.01).

### NLRP3 was involved in cell death induced by high glucose

**Figure 8:**
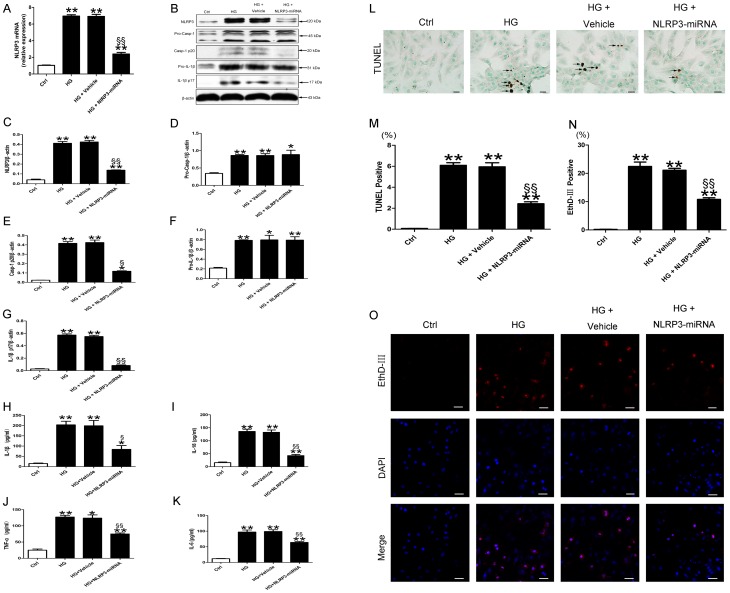
NLRP3 gene silencing suppressed activated caspase-1, inflammatory cytokines, and pyroptosis in high glucose-treated H9c2 cells. Relative mRNA expression of NLRP3 (A) and protein levels of NLRP3, pro-caspase-1, activated caspase-1, pro-IL-1β and mature IL-1β (B–G) in H9c2 cardiomyocytes with treatment. (H–K) The protein levels of IL-1β, IL-18, TNF-α and IL-6 in the cultured supernatants of H9c2 cardiomyocytes. (L) TUNEL staining of H9c2 cells, positive cell indicated by black arrow; scale bar: 20 µm. (M) Quantitative analysis of TUNEL positive cell in each group. (O) EthD-III (red) staining; scale bar: 50 µm. (N) Quantitative analysis of EthD-III positive cell. Data were presented as means±SEM, from 3 independent experiments. *p<0.05, **p<0.01 vs. Ctrl; ^§^p<0.05, ^§§^p<0.01 vs. HG+vehicle.

To explore the role of NLRP3 in high glucose-induced cell death of H9c2, we inhibited the expression of NLRP3 by lentivirial NLRP3-miRNA. The transfection efficacy of lentivirial NLRP3-miRNA in H9c2 cardiomyocytes reached 80% (Fig. S1D). Both the mRNA and protein levels of NLRP3 in cells transfected with NLRP3-miRNA were lower than the vehicle (Fig. 8A and B, both p<0.01). After inhibiting the expression of NLRP3, the protein levels of activated caspase-1 and mature IL-1β induced by high glucose decreased as compared with vehicle (Fig. 8E and G, p<0.05∼p<0.01). In addition, the secreted levels of IL-1β, IL-18, TNF-α and IL-6 in cultured supernatant of H9c2 cells were lower in HG+NLRP3-miRNA group than HG+vehicle (Fig. 8H–K, p<0.05∼p<0.01). NLRP3-miRNA did not have effect on the protein levels of pro-caspase-1 and pro-IL-1β (Fig. 8B, D and F). In keeping with these observations, the dead cell rate detected by TUNEL was lower in HG + NLRP3-miRNA than HG + vehicle (Fig. 8L and M, p<0.01). To avoid the interference of lentivector fluorescence, we did not use calcein AM in the detection of live cells. The high glucose induced-cell-death rate detected by EthD-III were decreased in NLRP3-miRNA treated cells as compared with vehicle (Fig. 8N and O, p<0.01).

### NF-κB and TXNIP mediated the ROS-induced NLRP3 inflammasome activation

**Figure 9:**
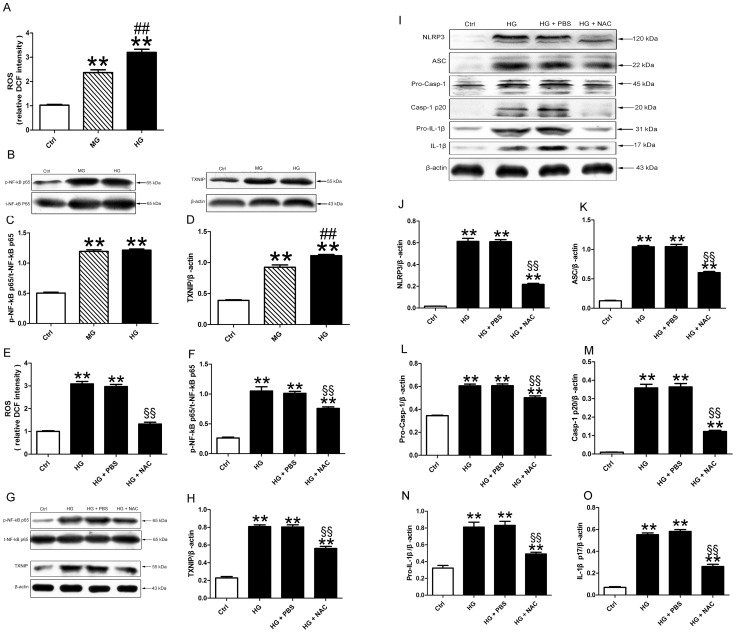
ROS induced NLRP3 inflammsome activation. (A) Quantitative analysis of the ROS production in H9c2 cells treated with medium and high glucose. (B–D) Western blot analysis of pNF-kB and TXNIP. (E) The production of cellular ROS after NAC treatment. The protein levels of pNF-kB (F and G), TXNIP (G and H), NLRP3 (I and J), ASC (I and K), pro-caspase-1 (I and L), activated caspase-1(I and M), pro-IL-1β (I and N) and mature IL-1β (I and O). Data were presented as means±SEM, from 3 independent experiments. **p<0.01 vs. Ctrl; ^##^p<0.01 vs. MG; ^§§^p<0.01 vs. HG+vehicle.

Glucose treatment promoted ROS generation in H9c2. The increase of ROS was dose dependent (Fig. 9A, all p<0.01). Coincidence with ROS production, the phosphorylation of NF-kB p65 was increased in medium and high glucose treated cells. And the expression of TXNIP also exhibited a dose-dependent response (Fig. 9B–D, all p<0.01). Pretreatment of cells with NAC inhibited high glucose-induced increase in intracellular ROS activity (Fig. 9E, p<0.01). Intriguingly, the levels of NF-kB phosphorylation and TXNIP were also lower in the NAC treated group than the PBS treated group (Fig. 9F–H, all p<0.01). In keeping with the decreased expression of NF-kB and TXNIP, the expression of all component of NLRP3, ASC, pro-caspaspe-1, activated caspase-1, pro- IL-1β and mature IL-1β were down-regulated with pretreatment of NAC as compared with PBS (Fig. 9I–O, all p<0.01).

**Figure 10:**
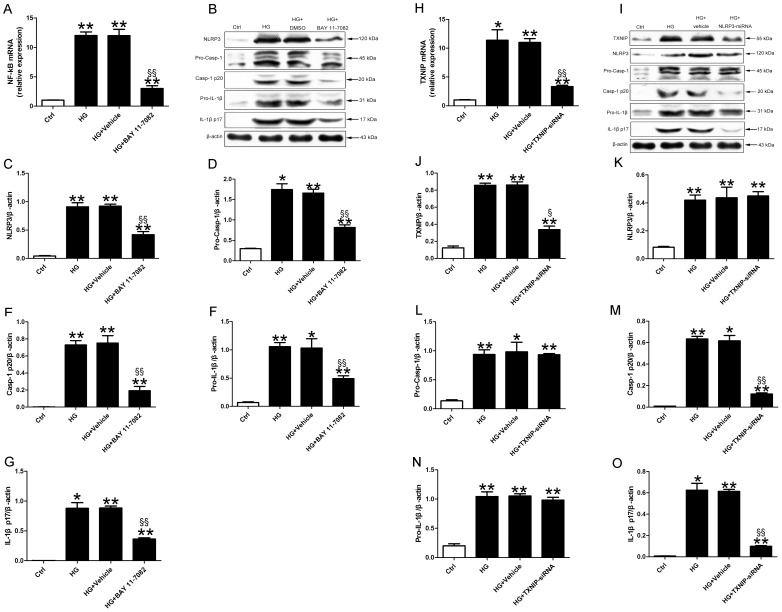
NF-kB and TXNIP induced NLRP3 inflammasome activation. Relative mRNA expression of NF-kB (A) and protein levels of NLRP3, pro-caspase-1, activated caspase-1, pro-IL-1β and mature IL-1β (B-G) in H9c2 cardiomyocytes with treatment. Relative mRNA expression of TXNIP (H) and protein levels of TXNIP, NLRP3, pro-caspase-1, activated caspase-1, pro-IL-1β and mature IL-1β (I-O) in H9c2 cardiomyocytes. Data were presented as means±SEM, from 3 independent experiments. *p<0.05, **p<0.01 vs. Ctrl; ^§§^p<0.01 vs. HG+vehicle.

Pretreatment of cells with NF-kB inhibitor BAY 11-7082 attenuated the mRNA expression of NF- kB. The protein levels of NLRP3 inflammasome, pro-IL-1β and mature IL-1β were decreased in HG+BAY 11-7082 group than in HG+DMSO group (Figure 10B–G, p<0.05∼p<0.01).

We inhibited the expression of TXNIP by TXNIP-siRNA plasmid. The transfection efficacy of TXNIP-siRNA plasmid into H9c2 cells reached 70% (Fig. S1E). Both the mRNA and protein levels of TXNIP in cells treated with TXNIP-siRNA were lower than the vehicle (Fig. 10H–J, p<0.05∼p<0.01). After inhibiting the expression of TXNIP, the protein levels of activated caspase-1 and mature IL-1β induced by high glucose decreased as compared with vehicle (Fig. 10I, M and O, both p<0.01). TXNIP-siRNA did not attenuate the protein levels of NLRP3, pro-caspase-1 and pro-IL-1β (Fig. 10I, K, L and N).

## Discussion

The present study provided new evidence to show the direct contribution of NLRP3 inflammasome in the pathogenesis of DCM, which was associated with the IL-1β-related metabolic disorder and caspase-1-mediated pyroptosis in myocardium. In addition, we found that the NF-kB and TXNIP were involved in the ROS-induced NLRP3 inflammasome activation. Thus, NLRP3 inflammasome has a pivotal role in DCM and could be an attractive drug target for treating type 2 diabetes.

In present study, we have successfully established type 2 diabetic rat models, which were characterized by severe metabolic disturbance, cardiac cell death, progressive myocardial dysfunction and remodeling. In keeping with previous studies [30], [31], we found early onset of diastolic dysfunction in diabetic rats at 8 weeks after diabetes in this study (data not shown). Intriguingly, the cardiac NLRP3 inflammasome and IL-1β expression in DM group were increased at 4 weeks, and reached to the highest levels at 8 or 12 weeks, and maintained at moderate high levels until 20 weeks of diabetes. The increase of NLRP3 inflammasome at early stage of diabetes was accompanied with the decrease in cardiac function in diabetic rats, which indicated the importance of NLRP3 inflammasome in the pathogenesis of DCM. The decrease of cardiac NLRP3 inflammasome in late stage of diabetes might be due to the adaptive protection of myocardium under the consistent stimuli in diabetes. Therefore, activation of NLRP3 inflammasome might contribute to the development and progression of DCM.

In light of the pivotal role of NLRP3 inflammasome in DCM, we used NLRP3-miRNA in vivo to explore whether downregulation of NLRP3 could prevent the pathogenesis of DCM. According to our preliminary study, we used 1×10∧^8^ TU/rat lentivirial vehicle or NLRP3-miRNA in this study. In our study, NLRP3-miRNA treatment produced no notable adverse effects and no deaths in rats. NLRP3 mRNA and protein levels were significantly decreased with NLRP3-miRNA treatment. Moreover, activated caspase-1 and mature IL-1β, the important effectors of NLRP3 inflammasome, were also significantly decreased in NLRP3-miRNA treated diabetic rats.

In current study, our diabetic rats showed severe metabolic disorders. However, the NLRP3 gene silencing therapy had little effect on improving these systemic metabolic disorders. Therefore, we could evaluate the effects of NLRP3 inflammasome on DCM without the influence of systemic changes.

In the diabetic state, induction of proinflammatory cytokines by hyperglycemia leads to the persistent inflammation in myocardium, which contributes to diabetic cardiac dysfunction [37]. Consistent with previous studies, we found an increased expression of IL-1β in the left ventricle of diabetes rats. The NLRP3-miRNA treatment effectively alleviated the cardiomyocyte inflammation by suppressing the release of IL-1β, IL-18, TNF-α and IL-6.

In addition to improving cardiac inflammation, NLRP3 silencing could reduce cardiac cell death in DCM. Cell death usually occurs through apoptosis or necrosis. The apoptosis of cardiomyocyte is an important pathological change, which causes loss of myocardial contractile unit and finally results in cardiac dysfunction in DCM [38]. Recent studies found that there are many other forms of cell death, one of which is pyroptosis [21], [23], [39], [40]. Pyroptosis, a proinflamatory form of programmed cell death, is caspase-1 dependent [17]. Pore formation of the plasma membrane, which presumably contributes to protein secretion at early stages of pyrotosis, can be the initiator of later cell death due to ruptures caused by cytoplasmic swelling [41]. Moreover, pyroptosis may lead to DNA damage in the nuleus [17]. Intriguingly, we found the pivotal characteristics of pyroptosis in myocardium of DCM in vivo, including activated caspase-1, cytoplasmic swelling, and nucleus DNA damage. These data suggested that pyroptosis participate in the pathogenesis of DCM. In vitro, the activated NLRP3 inflammasome lead to cytoplasmic caspase-1 activation, along with permeabilization of plasma membrane and nucleus DNA damage in high glucose-treated H9c2 cardiomyocytes. The result supported our conjecture that pyroptosis was present in cardiomyocyte. NLRP3 gene silencing in vivo and in vitro markedly suppressed caspase-1 activation, which was paralleled with decreased cell death characterized by integrity of cell membrane, cytoplamic swelling and DNA damage. Together, these data suggested that the caspase-1-dependent pyroptosis is a contributor to the hyperglycemia-induced cell death in DCM. NLRP3 was an important factor in the regulation of pyroptosis.

Our diabetic rats showed obvious lipid accumulation in the myocardium of DCM on TEM examination. The increased lipid droplets, which can induce cardiac lipotoxicity, is a contributor to the DCM [1], [31], [42]. After NLRP3 gene silencing, the cardiac lipid accumulation was markedly reversed.

Diabetic rats showed abnormal collagen deposition in interstitial areas on Masson and Sirius red staining. Intensive cardiac fibrosis, which can induce LV stiffness, is a common finding in progressed DCM [43]. Consistent with this, echocardiography revealed diastolic dysfunction. NLRP3 silencing reduced the aberrant interstitial collagen accumulation, collagen I and III content, and collagen I to III ratio in DCM. Furthermore, echocardiography results of NLRP3-miRNA treated diabetes rats revealed that both the LV systolic and diastolic dysfunction were improved, which were attributed to decreased cardiac cell death, reduced cardiac lipid accumulation, and less cardiac fibrosis; thus, NLRP3 silencing attenuated DCM.

Three distinct signal pathways have been proposed to be involved in NLRP3 inflammasome activation, including purinergic receptor P2X7, cathepsin B, and ROS related pathways [29]. NLRP3 activation can be blocked by ROS scavengers [44]. Additional levels of crosstalk between ROS and NLRP3 inflammasome may involve ROS-induced NF-kB activation of NLRP3 transcription, thereby facilitating the overexpression of NLRP3 inflammasome and maturation of caspase-1 and IL-1β [45], [46]. The activation of NLRP3 under oxidative stress also involves TXNIP, which can directly bind NLRP3 and induce NLRP3 to interact with ASC and caspase-1 [29], [47]. In line with these findings, our study showed glucose-treated H9c2 cardiomyocyte produced excessive ROS in a concentration-dependent manner. The levels of pNF-kB and TXNIP showed a similar expression pattern in response to glucose. With the ROS inhibition, the high glucose-induced NF-kB and TXNIP were markedly reduced. In keeping with these observations, the expression of NLRP3 inflammasome and mature IL-1β were decreased within ROS inhibition. Intriguingly, the ROS-induced NLRP3 inflammasome up-regulation was suppressed by NF-kB inhibitor, which indicated NF-kB was an essential link between ROS and NLRP3 inflammasome. One research of type 2 diabetes has shown that the maturation of caspase-1 and IL-1β, the sign of NLRP3 inflammation activation, did not required TXNIP in bone marrow-derived macrophages [48]. In contrast, some researches have indicated that TXNIP silencing could reduce NLRP3 inflammasome-mediated caspase-1 and IL-1β activation by affecting the TXNIP-NLRP3 interaction rather than decreasing the expression levels of NLRP3, pro-caspase-1 and pro-IL-1β [29], [49]. In our study, TXNIP-siRNA abrogated the maturation of caspase-1 and IL-1β without alteration of NLRP3 expression level in high glucose-treated H9c2 cardiomyocytes. The result indicated TXNIP was an important regulator of the activation of caspase-1 and IL-1β, which are the pivotal effecter of NLRP3 inflammasome.

## Conclusion

Our results suggest that the NLRP3 inflammasome is implicated in DCM. The activation of NLRP3 inflammasome and its effectors was regulated by NF-kB and TXNIP. Caspase-1 dependent pyroptosis had a distinct role in the process of DCM. NLRP3 gene silencing ameliorated the development of DCM in type 2 diabetic rat, by reducing cardiac inflammation, cardiomyocyte pyroptosis and left ventricular fibrosis. The cardioprotective effects with NLRP3 silencing suggest a potential role for NLRP3 antagonists in treating DCM in type 2 diabetes.

## Supporting Information

Figure S1 **Lentiviral vehicle and NLRP3-miRNA were effectively transfected in vivo and in vitro.** Mannose could not activate NLRP3 inflammsome. (A) Transfective efficiency of vehicle or NLRP3-miRNA in myocardium. Bright green points (white arrow) indicate GFP with lentivirus-NLRP3-miRNA or vehicle transfection (scale bar: 50 µm); n = 6. (B and C) Western blot analysis of NLRP3 inflammsome and IL-1β with 5.6 mM glucose or 27.5 mM mannose for 24 to 48 h; Data were presented as means±SEM, from 3 independent experiments. (D) Transfective efficiency of vehicle or NLRP3-miRNA in H9c2 cells. Bright green cells indicate GFP with lentivirus-NLRP3-miRNA or vehicle transfection (scale bar: 50 µm). (E) Transfective efficiency of vehicle or TXNIP-siRNA in H9c2 cells. Bright green cells indicate GFP with TXNIP-siRNA plasmid or vehicle transfection (scale bar: 50 µm).
(TIF)

## References

[1] Boudina S, Abel ED (2010) Diabetic cardiomyopathy, causes and effects. Rev Endocr Metab Disord 11: 31–39. 20180026 10.1007/s11154-010-9131-7 PMC2914514

[2] Falcao-Pires I, Leite-Moreira AF (2012) Diabetic cardiomyopathy: understanding the molecular and cellular basis to progress in diagnosis and treatment. Heart Fail Rev 17: 325–344. 21626163 10.1007/s10741-011-9257-z

[3] Cai L (2006) Suppression of nitrative damage by metallothionein in diabetic heart contributes to the prevention of cardiomyopathy. Free Radic Biol Med 41: 851–861. 16934665 10.1016/j.freeradbiomed.2006.06.007

[4] Cai L, Wang Y, Zhou G, Chen T, Song Y, et al (2006) Attenuation by metallothionein of early cardiac cell death via suppression of mitochondrial oxidative stress results in a prevention of diabetic cardiomyopathy. J Am Coll Cardiol 48: 1688–1697. 17045908 10.1016/j.jacc.2006.07.022

[5] Tsai KH, Wang WJ, Lin CW, Pai P, Lai TY, et al (2012) NADPH oxidase-derived superoxide anion-induced apoptosis is mediated via the JNK-dependent activation of NF-kappaB in cardiomyocytes exposed to high glucose. J Cell Physiol 227: 1347–1357. 21604272 10.1002/jcp.22847

[6] Devi TS, Lee I, Huttemann M, Kumar A, Nantwi KD, et al (2012) TXNIP links innate host defense mechanisms to oxidative stress and inflammation in retinal Muller glia under chronic hyperglycemia: implications for diabetic retinopathy. Exp Diabetes Res 2012: 438238. 22474421 10.1155/2012/438238 PMC3313582

[7] Bryant C, Fitzgerald KA (2009) Molecular mechanisms involved in inflammasome activation. Trends Cell Biol 19: 455–464. 19716304 10.1016/j.tcb.2009.06.002

[8] Lee HM, Kim JJ, Kim HJ, Shong M, Ku BJ, et al (2013) Upregulated NLRP3 inflammasome activation in patients with type 2 diabetes. Diabetes 62: 194–204. 23086037 10.2337/db12-0420 PMC3526026

[9] Wang C, Pan Y, Zhang QY, Wang FM, Kong LD (2012) Quercetin and allopurinol ameliorate kidney injury in STZ-treated rats with regulation of renal NLRP3 inflammasome activation and lipid accumulation. PLoS One 7: e38285. 22701621 10.1371/journal.pone.0038285 PMC3372527

[10] Franchi L, Munoz-Planillo R, Nunez G (2012) Sensing and reacting to microbes through the inflammasomes. Nat Immunol 13: 325–332. 22430785 10.1038/ni.2231 PMC3449002

[11] Schroder K, Zhou R, Tschopp J (2010) The NLRP3 inflammasome: a sensor for metabolic danger? Science 327: 296–300. 20075245 10.1126/science.1184003

[12] Schroder K, Tschopp J (2010) The inflammasomes. Cell 140: 821–832. 20303873 10.1016/j.cell.2010.01.040

[13] Lamkanfi M, Kanneganti TD (2010) Nlrp3: an immune sensor of cellular stress and infection. Int J Biochem Cell Biol 42: 792–795. 20079456 10.1016/j.biocel.2010.01.008 PMC2862759

[14] Bujak M, Frangogiannis NG (2009) The role of IL-1 in the pathogenesis of heart disease. Arch Immunol Ther Exp (Warsz) 57: 165–176. 19479203 10.1007/s00005-009-0024-y PMC2788964

[15] Coll NS, Epple P, Dangl JL (2011) Programmed cell death in the plant immune system. Cell Death Differ 18: 1247–1256. 21475301 10.1038/cdd.2011.37 PMC3172094

[16] Guarda G, So A (2010) Regulation of inflammasome activity. Immunology 130: 329–336. 20465574 10.1111/j.1365-2567.2010.03283.x PMC2913212

[17] Miao EA, Rajan JV, Aderem A (2011) Caspase-1-induced pyroptotic cell death. Immunol Rev 243: 206–214. 21884178 10.1111/j.1600-065X.2011.01044.x PMC3609431

[18] Lin J, Shou X, Mao X, Dong J, Mohabeer N, et al (2013) Oxidized low density lipoprotein induced caspase-1 mediated pyroptotic cell death in macrophages: implication in lesion instability? PLoS One 8: e62148. 23637985 10.1371/journal.pone.0062148 PMC3636212

[19] Fink SL, Bergsbaken T, Cookson BT (2008) Anthrax lethal toxin and Salmonella elicit the common cell death pathway of caspase-1-dependent pyroptosis via distinct mechanisms. Proc Natl Acad Sci U S A 105: 4312–4317. 18337499 10.1073/pnas.0707370105 PMC2393760

[20] Miao EA, Leaf IA, Treuting PM, Mao DP, Dors M, et al (2010) Caspase-1-induced pyroptosis is an innate immune effector mechanism against intracellular bacteria. Nat Immunol 11: 1136–1142. 21057511 10.1038/ni.1960 PMC3058225

[21] Lian LH, Milora KA, Manupipatpong KK, Jensen LE (2012) The double-stranded RNA analogue polyinosinic-polycytidylic acid induces keratinocyte pyroptosis and release of IL-36gamma. J Invest Dermatol 132: 1346–1353. 22318382 10.1038/jid.2011.482 PMC3326215

[22] Chung SD, Lai TY, Chien CT, Yu HJ (2012) Activating Nrf-2 signaling depresses unilateral ureteral obstruction-evoked mitochondrial stress-related autophagy, apoptosis and pyroptosis in kidney. PLoS One 7: e47299. 23071780 10.1371/journal.pone.0047299 PMC3468574

[23] Wree A, Eguchi A, McGeough MD, Pena CA, Johnson CD, et al.. (2013) NLRP3 inflammasome activation results in hepatocyte pyroptosis, liver inflammation and fibrosis. Hepatology. 10.1002/hep.26592 PMC4008151 23813842

[24] Shen X, Zheng S, Metreveli NS, Epstein PN (2006) Protection of cardiac mitochondria by overexpression of MnSOD reduces diabetic cardiomyopathy. Diabetes 55: 798–805. 16505246 10.2337/diabetes.55.03.06.db05-1039

[25] Van Linthout S, Spillmann F, Riad A, Trimpert C, Lievens J, et al (2008) Human apolipoprotein A-I gene transfer reduces the development of experimental diabetic cardiomyopathy. Circulation 117: 1563–1573. 18332268 10.1161/CIRCULATIONAHA.107.710830

[26] Yu W, Wu J, Cai F, Xiang J, Zha W, et al (2012) Curcumin alleviates diabetic cardiomyopathy in experimental diabetic rats. PLoS One 7: e52013. 23251674 10.1371/journal.pone.0052013 PMC3522633

[27] Bauernfeind FG, Horvath G, Stutz A, Alnemri ES, MacDonald K, et al (2009) Cutting edge: NF-kappaB activating pattern recognition and cytokine receptors license NLRP3 inflammasome activation by regulating NLRP3 expression. J Immunol 183: 787–791. 19570822 10.4049/jimmunol.0901363 PMC2824855

[28] Simard JC, Cesaro A, Chapeton-Montes J, Tardif M, Antoine F, et al (2013) S100A8 and S100A9 induce cytokine expression and regulate the NLRP3 inflammasome via ROS-dependent activation of NF-kappaB(1.). PLoS One 8: e72138. 23977231 10.1371/journal.pone.0072138 PMC3747084

[29] Zhou R, Tardivel A, Thorens B, Choi I, Tschopp J (2010) Thioredoxin-interacting protein links oxidative stress to inflammasome activation. Nat Immunol 11: 136–140. 20023662 10.1038/ni.1831

[30] Lacombe VA, Viatchenko-Karpinski S, Terentyev D, Sridhar A, Emani S, et al (2007) Mechanisms of impaired calcium handling underlying subclinical diastolic dysfunction in diabetes. Am J Physiol Regul Integr Comp Physiol 293: R1787–1797. 17761517 10.1152/ajpregu.00059.2007 PMC2413069

[31] Ti Y, Xie GL, Wang ZH, Bi XL, Ding WY, et al (2011) TRB3 gene silencing alleviates diabetic cardiomyopathy in a type 2 diabetic rat model. Diabetes 60: 2963–2974. 21933987 10.2337/db11-0549 PMC3198078

[32] Maeda H, Nagai H, Takemura G, Shintani-Ishida K, Komatsu M, et al (2013) Intermittent-hypoxia induced autophagy attenuates contractile dysfunction and myocardial injury in rat heart. Biochim Biophys Acta 1832: 1159–1166. 23499993 10.1016/j.bbadis.2013.02.014

[33] Liu X, Li B, Wang W, Zhang C, Zhang M, et al (2012) Effects of HMG-CoA reductase inhibitor on experimental autoimmune myocarditis. Cardiovasc Drugs Ther 26: 121–130. 22382902 10.1007/s10557-012-6372-6

[34] Luo B, Wang F, Li B, Dong Z, Liu X, et al (2013) Association of nucleotide-binding oligomerization domain-like receptor 3 inflammasome and adverse clinical outcomes in patients with idiopathic dilated cardiomyopathy. Clin Chem Lab Med 51: 1521–1528. 23382313 10.1515/cclm-2012-0600

[35] Liu L, Ding WY, Zhao J, Wang ZH, Zhong M, et al (2013) Activin receptor-like kinase 7 mediates high glucose-induced H9c2 cardiomyoblast apoptosis through activation of Smad2/3. Int J Biochem Cell Biol 45: 2027–2035. 23830891 10.1016/j.biocel.2013.06.018

[36] Dong Z, An F, Wu T, Zhang C, Zhang M, et al (2011) PTX3, a key component of innate immunity, is induced by SAA via FPRL1-mediated signaling in HAECs. J Cell Biochem 112: 2097–2105. 21465531 10.1002/jcb.23128

[37] Bell DS (2008) Diabetes: a cardiac condition manifesting as hyperglycemia. Endocr Pract 14: 924–932. 18996825 10.4158/EP.14.7.924

[38] Devereux RB, Roman MJ, Paranicas M, O'Grady MJ, Lee ET, et al (2000) Impact of diabetes on cardiac structure and function: the strong heart study. Circulation 101: 2271–2276. 10811594 10.1161/01.cir.101.19.2271

[39] McCoy AJ, Koizumi Y, Higa N, Suzuki T (2010) Differential regulation of caspase-1 activation via NLRP3/NLRC4 inflammasomes mediated by aerolysin and type III secretion system during Aeromonas veronii infection. J Immunol 185: 7077–7084. 21037094 10.4049/jimmunol.1002165

[40] Yang JR, Yao FH, Zhang JG, Ji ZY, Li KL, et al.. (2013) Ischemia/Reperfusion Induces Renal Tubule Pyroptosis via the CHOP-Caspase-11 Pathway. Am J Physiol Renal Physiol. 10.1152/ajprenal.00117.2013 24133119

[41] Fink SL, Cookson BT (2006) Caspase-1-dependent pore formation during pyroptosis leads to osmotic lysis of infected host macrophages. Cell Microbiol 8: 1812–1825. 16824040 10.1111/j.1462-5822.2006.00751.x

[42] van de Weijer T, Schrauwen-Hinderling VB, Schrauwen P (2011) Lipotoxicity in type 2 diabetic cardiomyopathy. Cardiovasc Res 92: 10–18. 21803867 10.1093/cvr/cvr212

[43] Asbun J, Villarreal FJ (2006) The pathogenesis of myocardial fibrosis in the setting of diabetic cardiomyopathy. J Am Coll Cardiol 47: 693–700. 16487830 10.1016/j.jacc.2005.09.050

[44] Dostert C, Petrilli V, Van Bruggen R, Steele C, Mossman BT, et al (2008) Innate immune activation through Nalp3 inflammasome sensing of asbestos and silica. Science 320: 674–677. 18403674 10.1126/science.1156995 PMC2396588

[45] Liao PC, Chao LK, Chou JC, Dong WC, Lin CN, et al (2013) Lipopolysaccharide/adenosine triphosphate-mediated signal transduction in the regulation of NLRP3 protein expression and caspase-1-mediated interleukin-1beta secretion. Inflamm Res 62: 89–96. 22986467 10.1007/s00011-012-0555-2

[46] Corsini E, Galbiati V, Nikitovic D, Tsatsakis AM (2013) Role of oxidative stress in chemical allergens induced skin cells activation. Food Chem Toxicol. 10.1016/j.fct.2013.02.038 23454144

[47] Martinon F (2010) Signaling by ROS drives inflammasome activation. Eur J Immunol 40: 616–619. 20201014 10.1002/eji.200940168

[48] Masters SL, Dunne A, Subramanian SL, Hull RL, Tannahill GM, et al (2010) Activation of the NLRP3 inflammasome by islet amyloid polypeptide provides a mechanism for enhanced IL-1beta in type 2 diabetes. Nat Immunol 11: 897–904. 20835230 10.1038/ni.1935 PMC3103663

[49] Mohamed IN, Hafez SS, Fairaq A, Ergul A, Imig JD, et al (2014) Thioredoxin-interacting protein is required for endothelial NLRP3 inflammasome activation and cell death in a rat model of high-fat diet. Diabetologia 57: 413–423. 24201577 10.1007/s00125-013-3101-z PMC3947289

