# Supplementary figures and images for: NLRP3 Gene Silencing Ameliorates Diabetic Cardiomyopathy in a Type 2 Diabetes Rat Model
Source: PLoS One. 2014 Aug 19;9(8):e104771. doi: 10.1371/journal.pone.0104771 (PMC4138036; doi:10.1371/journal.pone.0104771)

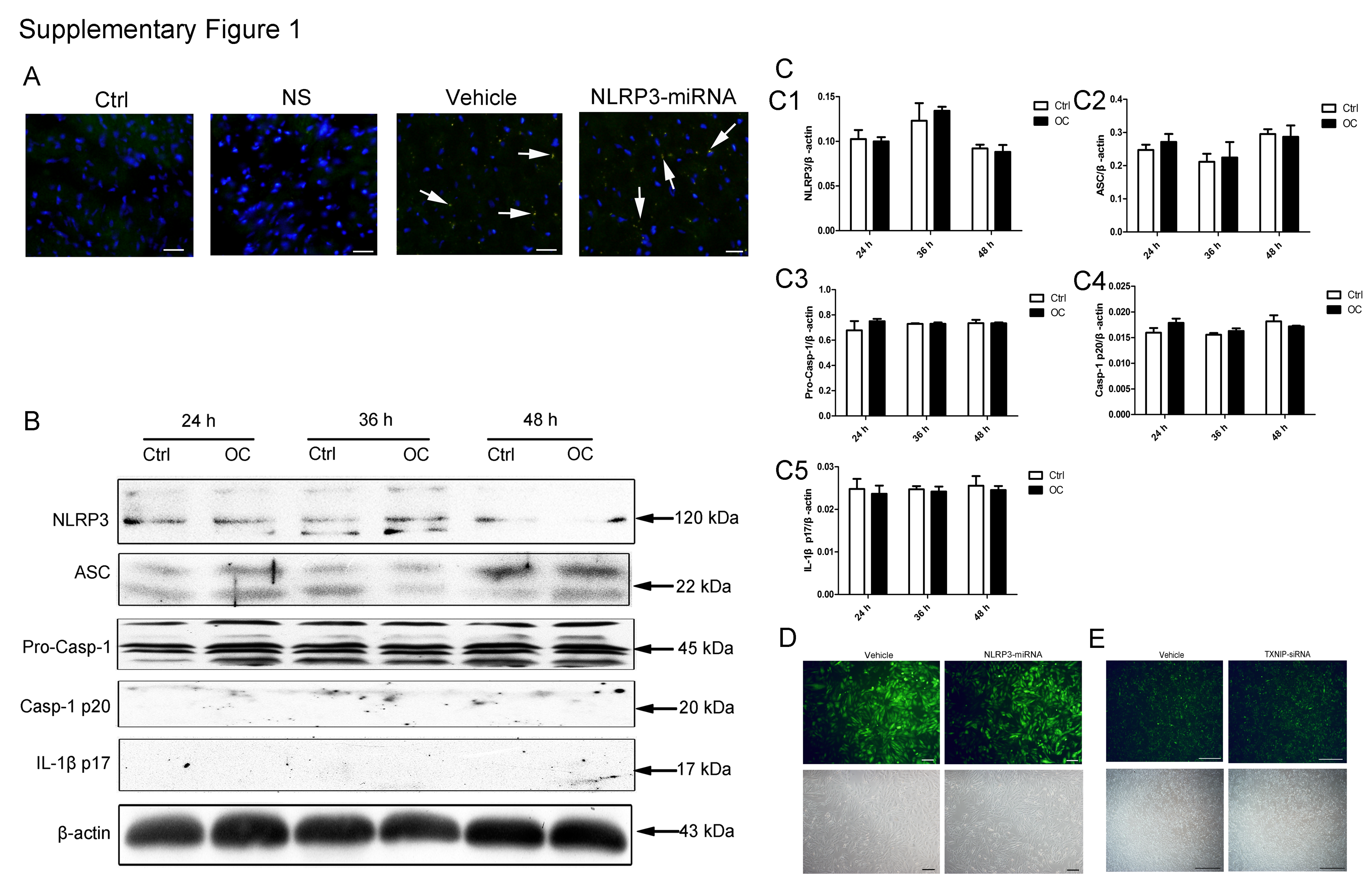

Supplement: Figure S1 — Lentiviral vehicle and NLRP3-miRNA were effectively transfected in vivo and in vitro. Mannose could not activate NLRP3 inflammsome. (A) Transfective efficiency of vehicle or NLRP3-miRNA in myocardium. Bright green points (white arrow) indicate GFP with lentivirus-NLRP3-miRNA or vehicle transfection (scale bar: 50 µm); n = 6. (B and C) Western blot analysis of NLRP3 inflammsome and IL-1β with 5.6 mM glucose or 27.5 mM mannose for 24 to 48 h; Data were presented as means±SEM, from 3 independent experiments. (D) Transfective efficiency of vehicle or NLRP3-miRNA in H9c2 cells. Bright green cells indicate GFP with lentivirus-NLRP3-miRNA or vehicle transfection (scale bar: 50 µm). (E) Transfective efficiency of vehicle or TXNIP-siRNA in H9c2 cells. Bright green cells indicate GFP with TXNIP-siRNA plasmid or vehicle transfection (scale bar: 50 µm). (TIF) [file pone.0104771.s001.tif]
